# Walking Psychotherapy As a Health Promotion Strategy to Improve Mental and Physical Health for Patients and Therapists: Clinical Open-Label Feasibility Trial

**DOI:** 10.1177/07067437211039194

**Published:** 2021-08-23

**Authors:** Nicole Koziel, Simone Vigod, Jennifer Price, Joanne Leung, Jennifer Hensel

**Affiliations:** 1 7985 Women’s College Hospital, Toronto, Ontario; 2 8664 University of Manitoba, Winnipeg, Manitoba

**Keywords:** post-traumatic stress disorder, walking, psychotherapy, mental health, exercise

## Abstract

**Background:**

Persons with mental illness are more at risk for sedentary behaviour and associated consequences. We assessed the feasibility of outdoor walking during psychotherapy sessions in an outpatient trauma therapy program to challenge sedentary behaviour.

**Methods:**

In this pilot trial in Toronto, Canada, female therapists and patients >18 years, were encouraged to walk during 12 consecutive trauma therapy sessions. Both groups were provided wearable pedometers. We assessed protocol feasibility and desirability, and 12-week changes in patient post-traumatic stress [PTSD check-list for DSM-5 (PCL-5)], and depression, anxiety, and stress symptoms [Depression, Anxiety and Stress Scale (DASS)].

**Results:**

91% (20/22) of patients approached for the study consented to participate and 17 (85%) completed follow-up questionnaires. There was walking in 132/197 (67%) of total therapy sessions (mean 7.3 out of 10.9 sessions per participant). Inclement weather was the predominant reason for in-office sessions. At 12-week follow-up, PCL-5 mean scores decreased from 38.4 [standard deviation, ((SD) 11.8) to 30.7 (SD 14.7)], [mean difference (MD) 7.7, 95% CI: 1.5 to 13.8]; 41% (7/17) participants had a clinically significant PCL-5 score reduction of >10 points. DASS-stress mean scores decreased from 19.0 to 16.0 (MD 3.0, 95% CI: 0.3 to 5.6). No changes were observed for DASS depression (MD -0.9, 95% CI: −5.1 to 3.3) nor DASS anxiety (MD -0.2, 95% CI: −3.1 to 2.7). Daily step reporting was inconsistent and not analyzed. There was high acceptability amongst patients and therapists to walk, but not to record daily steps. There were no adverse outcomes.

**Conclusions:**

It was feasible and acceptable to incorporate outdoor walking during trauma therapy sessions for patients and therapists. Weather was the greatest barrier to implementation. Further randomized-control study to compare seated and walking psychotherapy can clarify if there are psychotherapeutic and physical benefits with walking.

Childhood interpersonal trauma – exposure to events that threaten a child's physical and psychological safety – increases risk for mental illness and cardiovascular disease.^1^ Integrating walking into psychotherapy is a novel opportunity to improve cardiovascular risk factors and symptoms of mental illness, support emotion regulation, facilitate therapeutic alliance and integration of therapy skills, and habitualize walking.^2^ We evaluated the feasibility of integrating walking into outpatient trauma-focused psychotherapy in an open pilot trial.

We aimed to enroll 20 participants (age 18 +  years) receiving or waitlisted for individual psychotherapy in a hospital-based trauma therapy program in Toronto, Ontario with a predominantly female patient population (∼94%).^3^ Potentially eligible participants were sequentially approached for the study by participating MSW/RN/PhD/MD-trained therapists (five of the program’s 13 therapists participated, to target ∼4 participants/therapist). Research staff assessed eligibility. Individuals screening positive on the Physical Activity Readiness Questionnaire required medical clearance from their primary care providers. Weekly sessions followed Judith Herman's staged model of building safety and skills before processing traumatic memories in depth.^4^ While the therapy is not manualized, a relational therapy frame is followed by all program therapists to a maximum of 26 weekly 45−50 min sessions. Study sessions were held outdoors whenever possible, on well-maintained paths in a nearby city park. An advanced cardiovascular practice nurse led a 1-h therapist training session on safe walking practice prior to the study.

The primary outcome was protocol feasibility: recruitment and retention rates, proportion of sessions walked (and reasons not walked) and participant and therapist acceptability. The pilot was to be considered successful if 20 participants were recruited, and walking occurred the majority (>50%) of total sessions. The first five participants and four non-investigator therapists provided interim feedback via separate focus groups midway through the study. All participants and therapists were asked to provide narrative written feedback in response to specific questions at study completion. Clinical outcomes at 12-weeks post-enrollment were the PTSD checklist for DSM-5 (PCL-5) and Depression, Anxiety, and Stress Scale (DASS). A Fitbit Alta**^TM^** and daily step logs were provided to participants to record their activity. The Women's College Hospital Research Ethics Board approved the study (#2017-0040-B).

From 22 patients approached and meeting eligibility criteria from October 2017 to October 2018, 20 consented and 17 (85%) completed follow-up questionnaires. Mean age was 46.3 years (±10.6, 100% female), with clinically significant PTSD symptoms (mean PCL-5 = 39.5 ± 11.8) and mild-to-moderate mean DASS-depression (13.8 ± 8.0), DASS-stress (18.7 ± 9.2) and DASS-anxiety (8.8 ± 5.2) scores. About 60% (*n* = 12) had low-moderate baseline physical activity (International Physical Activity Questionnaire – short form). Walking occurred for 132 of 197 (67%) total therapy sessions. The mean number of sessions walked was 7.3 (range 3–11) out of 10.9 mean sessions per participant. Weather accounted for 35 (58%) of non-walking sessions, illness/injury for 15 (23%), and feeling too upset to walk for 5 (7.7%). Other reasons occurred <3 times (tired, late, needing to eat, paperwork review and therapist illness). Participant and therapist acceptability was high; the fluidity of the therapeutic frame introduced by walking outside the office setting was not felt to negatively impact progress. There was meaningful feedback – including that participants found daily step log completion burdensome – and no adverse events (Figure 1).


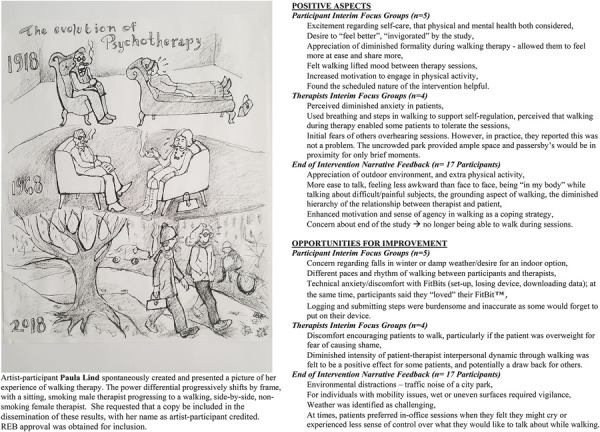


At 12-week follow-up (*n* = 17 participants), PCL-5 mean scores decreased from 38.4 (SD 11.8) to 30.7 (SD 14.7), [mean difference (MD) 7.7, 95% CI: 1.5 to 13.8]; 41% (7/17) participants had a clinically significant PCL-5 score reduction of >10 points. DASS-stress mean scores decreased from 19.0 to 16.0, (MD 3.0, 95% CI: 0.3 to 5.6). No changes were observed for DASS depression (MD -0.9, 95% CI: −5.1 to 3.3) nor DASS anxiety (MD -0.2, 95% CI: −3.1 to 2.7). Daily step reporting was inconsistent and not analyzed.

The study results suggest that walking during psychotherapy is well tolerated in this population, with symptom improvements in the desired direction. We could not quantitatively assess changes to participants’ overall level of physical activity, but participants qualitatively reported an increase in non-sedentary behaviour. These results are consistent with those of a small study of older, hospitalized patients with depression,^5^ but minimal research has been done in this area for ambulatory patients, so the current study is novel. Limitations were the all-female participant and therapist population, high baseline activity levels of 40% of participants and that one investigator was an intervention-provider. Generalizability to settings where a proximate, safe, outdoor walking space is not as readily available is a consideration and options for indoor walking are likely needed for cold and rainy climates. Finally, with a small sample size and no control group, the focus was on feasibility of the model and not efficacy for mental and physical outcomes, including how the depth of the therapy compares to that of office-based settings. Incorporating this pilot's learnings, a future randomized controlled trial could aim to answer these important questions.
